# Primary Thyroid Lymphoma: A Case Series

**DOI:** 10.7759/cureus.89197

**Published:** 2025-08-01

**Authors:** Yousef Alalawi, Tahani N Alrashidi, Sarah Shafie, Laila Moharram, Abeer Asiri

**Affiliations:** 1 Department of Surgery, King Salman Armed Forces Hospital, Tabuk, SAU; 2 Department of Histopathology, King Salman Armed Forces Hospital, Tabuk, SAU; 3 Department of Surgery, King Khalid Hospital, Tabuk, SAU

**Keywords:** chemotherapy (ct), core needle biopsy, hashimoto's thyroiditis, primary thyroid lymphoma (ptl), radiotherapy (rt)

## Abstract

Background

Primary thyroid lymphoma (PTL) is a rare form of thyroid cancer. It is frequently accompanied by Hashimoto's thyroiditis. Treatment approaches differ significantly from those used for other types of thyroid cancer. This study aimed to examine the clinical presentation, diagnostic methods, and treatment strategies employed in patients with PTL.

Materials and methods

We present a retrospective case series of seven patients diagnosed with PTL at a tertiary care hospital between 2017 and 2021.

Results

Our sample included seven patients, five (71.4%) females and two (28.6%) males. The mean age was 57.8, and their ages ranged from 39 to 77. Four of our patients were diagnosed with Hashimoto's thyroiditis. Most of the patients presented with a rapidly enlarging mass associated with compression symptoms. The diagnosis of PTL was made by core needle biopsy and surgical excision. They all had diffuse large B-cell lymphoma (DLBCL) in the histopathological examination. Out of the total patients, 28.6% (two patients) experienced mortality: one in stage III and one in stage IV.

Conclusion

Any patient presenting with a rapidly growing thyroid mass accompanied by a history of Hashimoto's thyroiditis should raise the possibility of PTL. In our retrospective case series, DLBCL was identified as the predominant subtype. Fine-needle aspiration cytology (FNAC) demonstrated limited diagnostic modalities, with none of the cases achieving a definitive diagnosis. In contrast, core needle biopsy was the preferred diagnostic tool. Surgery plays a limited role and is mainly for diagnosis. Prognosis depends on the stage of the disease at diagnosis.

## Introduction

Primary thyroid lymphoma (PTL) is a rare type of tumor that originates in the thyroid gland [1]. It accounts for 1-5% of all thyroid malignancies and less than 2% of extranodal lymphomas [1]. The two main histological types of thyroid lymphoma are diffuse large B-cell lymphoma (DLBCL) and mucosa-associated lymphoid tissue (MALT) lymphoma. Non-Hodgkin's lymphoma is the most common type of PTL (98%) [2]. PTL is associated with Hashimoto's thyroiditis, with several studies suggesting that up to 80% of patients with PTL have a background of Hashimoto's thyroiditis [2,3]. It usually presents as a rapidly growing painless mass in the thyroid glands, leading to compression symptoms such as difficulty swallowing and hoarseness [2,4]. PTL commonly progresses in a short period, over weeks to months, and up to 10-14% of patients have systemic "B" symptoms such as fever, night sweats, and weight loss [5,6].

Diagnosis of PTL overlaps with other thyroid malignancies, such as anaplastic thyroid carcinoma, making diagnosis difficult [7]. The diagnosis is usually made through either fine-needle aspiration cytology (FNAC) or biopsy, as PTL is difficult to distinguish from other malignancies or benign processes by radiological images [4].

The management and prognosis of PTL differ depending on the histological type and stage of disease at diagnosis. The treatment modalities are either surgical intervention, radiotherapy, chemotherapy, or their combination [8]. DLBCL, the most aggressive and common subtype, requires chemotherapy, combined with radiotherapy. The R-CHOP regimen (rituximab, cyclophosphamide, doxorubicin, vincristine, and prednisone), commonly used in DLBCL, significantly improved survival outcomes [9]. Compared to other thyroid cancers, surgical intervention is generally limited in PTL and is primarily reserved for diagnostic purposes or to relieve compressive symptoms [2].

Due to low disease incidence, several aspects of PTL remain poorly understood, and current knowledge depends on retrospective case series, small cohort studies, and institutional reviews. Furthermore, the overlap in presentation between PTL and other thyroid malignancies often leads to a delay in diagnosis, which can adversely affect outcomes [10]. This report presents seven cases of PTL diagnosed and managed at our institution.

## Materials and methods

Study design and setting

We present a retrospective case series of seven patients diagnosed with PTL based on histopathological diagnosis at King Salman Armed Forces Hospital (KSAFH) in Tabuk, Saudi Arabia, from 2017 to 2021.

Data collection 

Data was collected retrospectively from medical records. Variables collected included age, gender, past medical history, surgical history, imaging studies, diagnostic modalities, histopathological findings, management modalities, and clinical presentation: compression symptoms include difficulty swallowing, shortness of breath, and changes in voice or breathing patterns, while B symptoms include fever, night sweats, and weight loss.

Inclusion and exclusion criteria

Patients who met the inclusion criteria for this study were diagnosed and treated at KSAFH. Patients who received management or were diagnosed outside our institution or had incomplete medical records that limited comprehensive evaluation were excluded from the final dataset.

Ethical considerations

Ethical approval was obtained from the Research Ethics Committee of KSAFH (approval number: KSAFH-RET-2024-587).

Statistical analysis

Data were evaluated using IBM SPSS Statistics for Windows, Version 20.0 (Released 2019; IBM Corp., Armonk, New York, United States). Quantitative data were described as means and ranges, while qualitative data were expressed as frequencies and percentages.

## Results

Seven patients were diagnosed with PTL between 2017 and 2021: five (71.4%) females and two (28.6%) males. The mean age was 57.8, and their ages ranged from 39 to 77. Most patients (six, 85.7%) presented with a suddenly growing mass in the neck (Table 1).

**Table 1:** Clinical features

Clinical feature	n	%
Rapidly enlarging neck mass	6	85.7%
B symptoms	1	14.2%
Compression symptoms	4	57.1%

**Figure 1:**
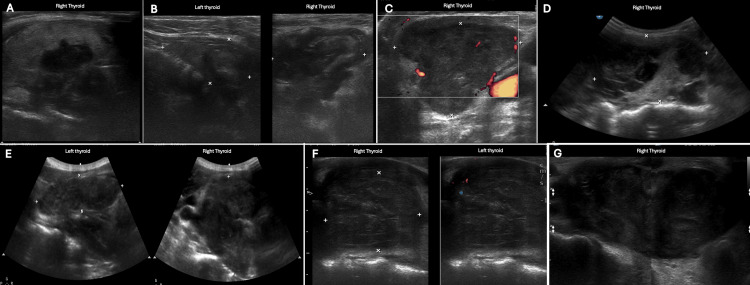
Ultrasound findings (A) The right is almost totally replaced by a large heterogeneous, hypoechoic lesion with evident vascularity and irregular outline. (B) Diffuse enlargement of the thyroid gland with ill-defined margins and possible retrosternal extension, showing heterogeneous echotexture with multiple variable-sized nodules. (C) Heterogeneous mass lesion, mainly hypoechoic, occupying most of the right thyroid lobe and showing minimal internal color flow. (D) Enlarged right lobe of the thyroid gland noted with a complex cystic nodule having internal echoes. (E) Both thyroid lobes are diffusely markedly enlarged and heterogeneous with multiple hypoechoic small nodules. (F) Large heterogeneous nodule seen occupying and enlarging the left thyroid gland. (G) Enlarged right thyroid lobe and isthmus, nearly totally occupied by a large heterogeneous hypoechoic nodule.

Ultrasound was done as an initial investigation of all patients (Figure 1).

Thyroid function tests showed that three patients (43%) were euthyroid and four (57%) were hypothyroid. FNAC had been performed before histopathological evaluation in all our patients; unfortunately, none were diagnostic. Only four (57%) patients were suspicious of lymphoma. Three of our patients were diagnosed through histopathological examination after surgery. Four were diagnosed by core needle biopsy (CNB). 

One patient underwent total thyroidectomy alone (refused chemotherapy and radiation therapy (RT); still alive), and one underwent total thyroidectomy with R-CHOP. One patient underwent total thyroidectomy with R-CHOP and involved site radiation therapy (ISRT). Three patients received R-CHOP and ISRT. One patient underwent a tracheostomy to secure the airway, and she refused chemotherapy and radiotherapy (Table 2).

**Table 2:** Case series table: demographics, diagnosis, and management HT: Hashimoto's thyroiditis; FNAC: fine-needle aspiration cytology; CNB: core needle biopsy (Tru-cut biopsy); DLBCL: diffuse large B-cell lymphoma; R-CHOP: rituximab, cyclophosphamide, doxorubicin, vincristine, and prednisone; ISRT: involved site radiation therapy; CR: complete response; DM: diabetes mellitus; HTN: hypertension; TT: total thyroidectomy; LN: lymph node; PTC: papillary thyroid cancer; BC: breast cancer; MRM: modified radical mastectomy; CT: chemotherapy; RT: radiation therapy

Cases	Age	Past medical/surgical history	Diagnosis	Histopathology	Treatment	Stage	Status at the last visit
1	50	HT	FNAC suspected, CNB	DLBCL	R-CHOP, ISRT	I	CR, alive
2	55	HT, DM, HTN	Refused FNAC and CNB, TT with LN dissection	DLBCL, PTC	TT, R-CHOP, ISRT	II	CR, alive
3	77	HT, DM, HTN, BC, post-MRM	Refused FNAC and CNB, TT	DLBCL	TT, refused CT and RT	I	Alive
4	48	HTN, smoker	FNAC suspected, CNB	DLBCL	R-CHOP, tracheostomy, ISRT	II	CR, alive
5	39	None	FNAC suspected, CNB	DLBCL	R-CHOP, ISRT	II	CR, alive
6	61	DM, HT	Refused FNAC and CNB, TT	DLBCL	TT, R-CHOP	III	Dead
7	75	HTN	FNAC suspected, CNB	DLBCL	Tracheostomy, refused CT and RT	IV	Dead

**Figure 2:**
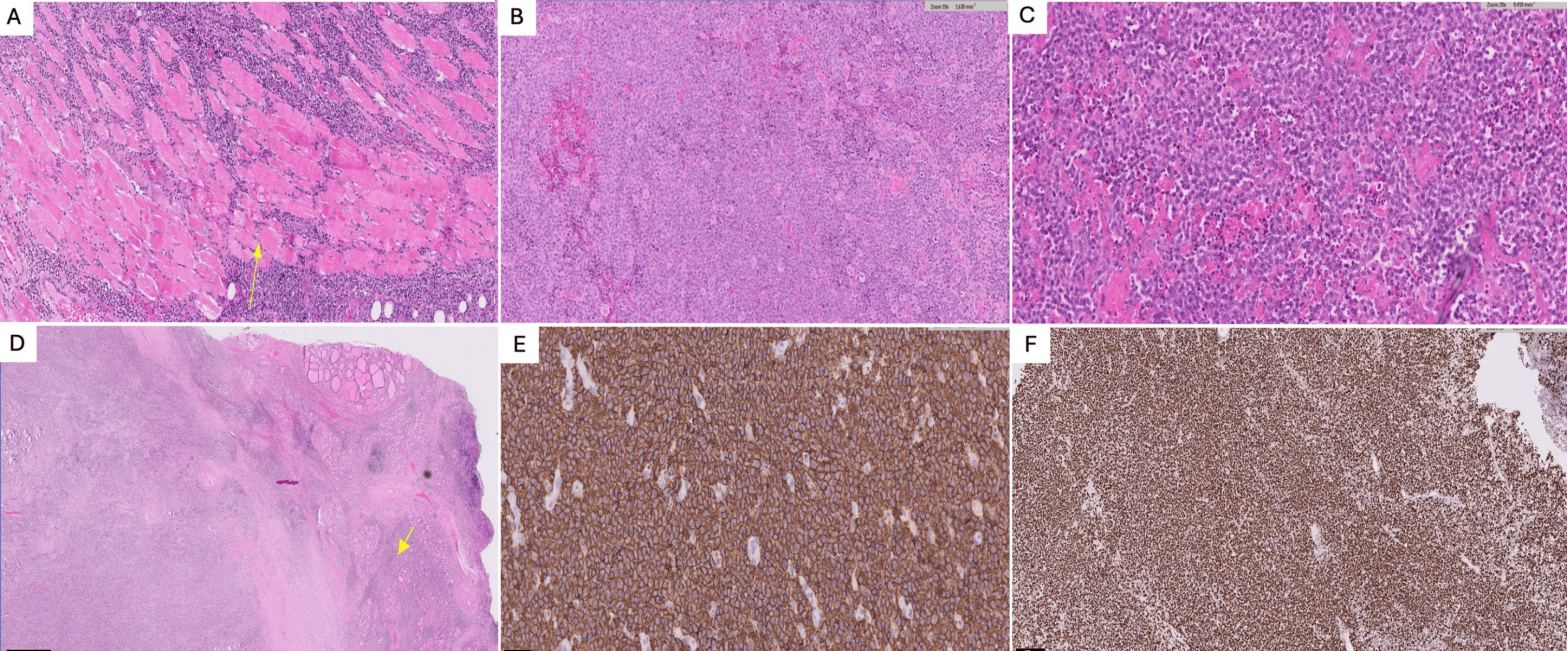
Histopathology selected images of cases (A) The tumor infiltrates the skeletal muscle fibers (arrow), H&E stain. (B) The tumor is composed of sheets of large cells. No more thyroid follicles are seen in this field, H&E stain. (C) The tumor shows areas of necrosis and numerous apoptotic bodies. (D) The background thyroid shows lymphocytic thyroiditis (arrow), H&E stain. (E) Tumor cells are positive for the B-cell marker CD20. (F) This case showed high Ki-67 proliferation index (more than 90% of nuclei are positive).

Histopathological examination revealed DLBCL in all patients (Figure 2).

Four (57%) had a non-germinal center phenotype, while three (43%) had a germinal center phenotype.

According to the Ann Arbor staging system, the following were the classifications of the cases: two cases, stage IE; three cases, stage IIE; one case, stage III; and one case, stage IV. Bone marrow biopsy (BMB) showed normal cellularity in three cases, while it was not done in four cases. The extension was evaluated in all patients with computed tomography scans, as well as BMB. Four patients (57%) had a past medical history of Hashimoto's thyroiditis. Out of the total patients, 28.6% (two patients) experienced mortality: one in stage III and one in stage IV (Table 2).

## Discussion

The predominant histological subtype of PTL is DLBCL, which is characterized by aggressive disease progression and a tendency to be detected at advanced stages [2]. PTL is more commonly detected in females and exhibits a 2-8 times higher prevalence in women than in men [2,11]. In the present study, five of the seven patients were women. 

Although the etiology of PTL remains unclear, many patients with DLBCL have a history of thyroid disease. Longstanding Hashimoto's thyroiditis often acts as a common predisposing factor, with an increasing risk, 40-80 times more than that in the general population [2]. In our study, four of the seven patients were diagnosed with Hashimoto's thyroiditis.

Diagnosis of PTL is primarily based on imaging features and is confirmed histopathologically [2]. Ultrasound is considered the initial diagnostic modality in patients who are suspected or confirmed to have thyroid cancer [12]. PTL is suspected based on clinical manifestations and ultrasonography findings. The next step was to confirm the diagnosis by histological examination using either biopsy or FNAC [13].

In the literature, FNAC sensitivity varies depending on the expert pathologist, as reported by Agarwaf et al. [14]. FNAC accurately diagnosed PTL in only 60% of patients who were later found to have malignancy, whereas in another study from India, 90% of PTL cases were correctly diagnosed using FNAC [14]. In our study, FNAC was performed in all patients; however, none were diagnosed, and only four patients were suspected to have lymphoma. In a study by Acar et al., half of the patients were diagnosed using FNAC, and half were diagnosed after surgical excision [1].

Several studies have reported that FNAC has low sensitivity for the diagnosis of PTL (50-80%) and is most often diagnostic in patients with a large lymphoma burden [13-17]. However, the role of FNAC in distinguishing between different diagnoses such as Hashimoto's thyroiditis, lymphocytic thyroiditis, anaplastic carcinoma thyroid, and low-grade lymphoma is insufficient. Thus, a CNB, surgical incisional biopsy, or even thyroidectomy is usually needed to reach a diagnosis [13-16]. In our study, three patients were diagnosed by histopathological examination after surgery. Four patients were diagnosed using Tru-cut biopsy (CNB). 

As CNB provides more tissue for analysis, it is considered the best method for diagnosing PTL and differentiating between PTL and anaplastic carcinoma. CNB has a sensitivity of 95% and is useful for lymphoma subtyping [2]. Currently, surgical interventions are only recommended when other invasive investigations fail to reach a diagnosis [13-18]. 

The sensitivity of CNB is higher than FNAC. In CNB, there are more adequate tissue samples, and maintaining tissue architecture helps differentiate between thyroiditis and lymphoma and determine the lymphoma subtype. Although FNAC has improved with newer modalities, such as cytometry and immunohistochemical staining, which help establish the monoclonality of lymphocytes, CNB remains suggested for definitive diagnosis when PTL is suspected [13,19]. In a systematic review and meta-analysis done by Goedseels et al., which included 166 patients, the sensitivity of CNB for diagnosing PTL was reported to be 93.8%. Furthermore, the rate of diagnostic surgery after CNB is only 6.2% [20]. In our study, one patient was diagnosed with PTL coexisting with papillary thyroid carcinoma. This is similar to the results reported by Acar et al. [1]. Therefore, PTL may be associated with other primary thyroid malignancies, such as papillary, follicular, and anaplastic cancers. Thus, in addition to histopathological analysis, it is important to perform immunohistochemical staining with monoclonal antibodies [1]. 

According to the Ann Arbor staging system, in our study, most patients (71%) were diagnosed with stage IE or IIE disease. Similar to previous studies, most patients were diagnosed in the early stages [2,4].

PTL requires a multidisciplinary approach and an aggressive clinical course; treatment modalities such as surgery, radiotherapy, chemotherapy, or a combination of these; and, recently, the introduction of immunotherapy [6,12,13].

Traditionally, surgery and radiotherapy have been used to treat local neoplasms. Chemotherapy is an essential treatment for metastatic disease [2]. DLBC is sensitive to radiotherapy and chemotherapy, particularly to the combination of cyclophosphamide, doxorubicin, vincristine, and prednisone (CHOP) chemotherapy and the monoclonal antibody rituximab, which targets CD20 [2]. 

The surgical intervention in PTL, especially aggressive surgery, is currently not recommended as it increases the risk of morbidities and damage to the recurrent laryngeal nerves, trachea, esophagus, and large vessels as well as offers no significant advantage compared to cases that received radiotherapy, chemotherapy, and biopsy [2]. In cases where less invasive modalities fail to achieve a diagnosis, surgical intervention is useful [2]. In our study, we performed total thyroidectomy in three patients to relieve compression symptoms and achieve the diagnosis. 

A previous study showed that four of seven patients underwent surgery alone or were followed by radiotherapy [15]. However, although surgical intervention is useful, particularly in patients with compressive symptoms or patients who underwent palliative treatment, to protect the airway, use of minimally invasive and temporary interventions such as tracheostomy and corticosteroids to secure the airway is preferred [4,15]. 

In our study, one patient underwent total thyroidectomy alone (patient refused CT and RT; still alive), and one underwent total thyroidectomy with R-CHOP. One patient underwent total thyroidectomy with R-CHOP and ISRT. Three patients received R-CHOP and ISRT. One patient underwent tracheostomy to secure the airway; she refused chemotherapy and radiotherapy (Table 2).

A recent study in 2020 suggested that chemotherapy has good results in stages I and II for small neoplasms less than 5 cm [2]. Radiation therapy is used to consolidate the response to CHOP. In addition, rituximab is taken as part of the CHOP regimen (R-CHOP) [2].

This study is limited by its small sample size; its retrospective, single-center design; and incomplete staging in three patients due to refusal of BMB, which limited the ability to evaluate bone marrow involvement.

## Conclusions

PTL remains a rare and challenging disease. Patients who present with a rapidly enlarging neck mass with underlying Hashimoto's thyroiditis should raise the possibility of PTL. In this retrospective case series, DLBCL was identified as the predominant subtype. FNAC demonstrated limited diagnostic modalities, with none of the cases achieving a definitive diagnosis. CNB, in contrast, provided sufficient tissue architecture for accurate histopathological and immunophenotypic evaluation, supporting its role as a preferred diagnostic modality.

Most patients were diagnosed at early stages (IE and IIE) and responded to combined chemo-radiotherapy. Mortality is higher in advanced-stage disease. Further prospective research, multi-center studies are necessary to establish standardized diagnostic modalities and effective management in patients with PTL.
